# Enhanced *Leishmania braziliensis* Infection Following Pre-Exposure to Sandfly Saliva

**DOI:** 10.1371/journal.pntd.0000084

**Published:** 2007-11-28

**Authors:** Tatiana R. de Moura, Fabiano Oliveira, Fernanda O. Novais, José Carlos Miranda, Jorge Clarêncio, Ivonise Follador, Edgar M. Carvalho, Jesus G. Valenzuela, Manoel Barral-Netto, Aldina Barral, Cláudia Brodskyn, Camila I. de Oliveira

**Affiliations:** 1 Centro de Pesquisas Gonçalo Moniz, Fundação Oswaldo Cruz (FIOCRUZ), Salvador, Brazil; 2 Vector Molecular Biology Unit, National Institute of Allergy and Infectious Diseases, National Institutes of Health, Rockville, Maryland, United States of America; 3 Universidade Federal da Bahia, Salvador, Brazil; 4 Instituto de Investigação em Imunologia, Salvador, Brazil; Hebrew University, Israel

## Abstract

**Background:**

Sand fly saliva has an array of pharmacological and immunomodulatory components, and immunity to saliva protects against *Leishmania* infection. In the present study, we have studied the immune response against *Lutzomyia intermedia* saliva, the main vector of *Leishmania braziliensis* in Brazil, and the effects of saliva pre-exposure on *L. braziliensis* infection employing an intradermal experimental model.

**Methodology/principal findings:**

BALB/c mice immunized with *L. intermedia* salivary gland sonicate (SGS) developed a saliva-specific antibody response and a cellular immune response with presence of both IFN-γ and IL-4. The inflammatory infiltrate observed in SGS-immunized mice was comprised of numerous polymorphonuclear and few mononuclear cells. Mice challenged with live *L. braziliensis* in the presence of saliva were not protected although lesion development was delayed. The inoculation site and draining lymph node showed continuous parasite replication and low IFN-γ to IL-4 ratio, indicating that pre-exposure to *L. intermedia* saliva leads to modulation of the immune response. Furthermore, in an endemic area of cutaneous leishmaniasis, patients with active lesions displayed higher levels of anti-*L. intermedia* saliva antibodies when compared to individuals with a positive skin test result for *Leishmania*.

**Conclusion:**

These results show that pre-exposure to sand fly saliva plays an important role in the outcome of cutaneous leishmaniasis, in both mice and humans. They emphasize possible hurdles in the development of vaccines based on sand fly saliva and the need to identify and select the individual salivary candidates instead of using whole salivary mixture that may favor a non-protective response.

## Author Summary

Parasites of the genus *Leishmania* cause a variety of diseases known as leishmaniasis, that are transmitted by bites of female sand flies that, during blood-feeding, inject humans with parasites and saliva. It was shown that, in mice, immunity to sand-fly saliva is able to protect against the development of leishmaniasis. We have investigated, in the present study, whether this finding extends the sand fly species *Lutzomyia intermedia,* which is responsible for transmission of *Leishmania braziliensis,* a parasite species able to cause destructive skin lesions that can be fatal if left untreated. We observed that mice injected with sand fly saliva develop a specific immune response against salivary proteins. Most importantly, however, this immune response was unable to protect mice against a challenge infection with *L. braziliensis*, indicating that exposure to this sand fly saliva is harmful to the host. Indeed, subjects with cutaneous leishmaniasis have a higher immune response against *L. intermedia* saliva. These findings indicate that the anti-saliva immune response to sand fly saliva plays an important role in the outcome of leishmaniasis caused by *L. braziliensis*, in both mice and humans, and emphasize possible hurdles in the development of vaccines based on sand fly saliva.

## Introduction

Protozoan parasites of the genus *Leishmania* cause a broad spectrum of diseases, collectively known as leishmaniasis, that occur predominantly in tropical and subtropical regions. The leishmaniases are transmitted by different species of sand flies, and depending on the *Leishmania* species involved and the genetic makeup or immunological status of the host, different clinical manifestations of the disease are observed.

The mammalian host acquires leishmaniasis when it is bitten by an infected sand fly vector. During parasite inoculation, the host is also injected with the sand fly saliva which has been shown to contain a repertoire of bio-active molecules capable of interfering with the host's hemostatic, inflammatory and immune responses (reviewed in [1],[2],[3]). Among the latter, it has been shown that sand fly saliva can exert a direct effect upon the function of cells from the immune system [4],[5],[6]. In fact, it was shown early on that co-inoculation of *L. longipalpis* or *P. papatasi* salivary gland sonicate (SGS) and *L. major* led to a significant exacerbation of lesion size and parasite load in BALB/c mice [7],[8]. Similar effects were observed with *L. braziliensis* [9],[10] and *L. amazonensis* [11].

On the other hand, mice are protected against *L. major* when immunized with SGS [8], when pre-exposed to the bites of uninfected sand flies [12] or, more recently, when immunized with saliva components [13],[14]. Since the composition of salivary molecules, their function and antigenicity varies among distinct sand fly species [15],[16],[17] it is of foremost importance to investigate whether vector-based vaccines can also be developed against other *Leishmania* species. American cutaneous leishmaniasis, caused by *Leishmania braziliensis*, is distinguished from other leishmaniases by its chronicity, latency and tendency to metastasize in the human host leading to muco-cutaneous leishmaniasis [18],[19]. In this sense, we have, in the present report, investigated (a) the immunogenic properties of *L. intermedia* saliva, the main vector of *L. braziliensis* in Brazil, (b) the effect of vaccination with *L. intermedia* saliva followed by challenge with *L. braziliensis* plus saliva and c) anti-saliva immune response of individuals from an endemic area of cutaneous leishmaniasis. In order to do so, we employed a recently developed experimental model of cutaneous leishmaniasis (CL), based upon the inoculation of *L. braziliensis* parasites into the dermis of BALB/c mice [20].

## Materials and Methods

### Mice

Female BALB/c mice (6–8 weeks of age) were obtained from CPqGM/FIOCRUZ Animal Facility where they were maintained under pathogen-free conditions. All procedures involving animals were approved by the local Ethics Committee on Animal Care and Utilization (CEUA - CPqGM/FIOCRUZ).

### Sand Flies and Preparation of SGS

*Lutzomyia intermedia*, Corte de Pedra strain, and *Lutzomyia longipalpis*, Cavunge strain, were reared at Centro de Pesquisas Gonçalo Moniz-FIOCRUZ, as described elsewhere [21]. Adult sand flies were used for dissection of salivary glands at 3–5 days after emergence. Salivary glands were stored in groups of 20 pairs in 20 µl NaCl (150 mM) Hepes buffer (10 mM, pH 7.4), at −70°C. Immediately before use, salivary glands were disrupted by ultrasonication within 1.5 ml conical tubes. Tubes were centrifuged at 10,000×g for 2 min and the resultant supernatant (Salivary Gland Sonicate - SGS) was used for the studies. The level of LPS contamination of SGS preparations was determined using a commercially available LAL Chromogenic Kit (QCL-1000, Lonza Bioscience); LPS concentration was <0.1 ng/ml and SGS did not stimulate human monocytes. In order to evaluate the immunogenic potential of *L. intermedia* saliva, BALB/c mice were immunized three times with SGS (equivalent to 1 pair of salivary glands) in 10 µl of PBS, in the dermis of the right ear, using a 27.5 G needle. Immunizations were performed at 2 week intervals. Control mice were injected with PBS.

### Analysis of anti-saliva antibodies by ELISA and Western blot

ELISA microplates were coated overnight at 4°C with 50 µl SGS diluted to 5 pairs of salivary glands/ml in coating buffer (NaHCO_3_ 0.45 M, Na_2_CO_3_ 0.02 M, pH 9.6). After washing with PBS-Tween, wells were blocked with PBS-Tween plus 5% dried skim milk for 1 hour at 37°C. Wells were incubated overnight with sera from control or immunized mice, obtained two weeks after the last immunization, diluted (1∶50) in PBS-Tween. After further washings, wells were incubated with alkaline phosphatase-conjugated anti mouse IgG antibody (Promega) diluted (1∶5000) in PBS-Tween, for 1 hour at 37°C. Following another washing cycle, wells were developed with p-nitrophenylphosphate in sodium carbonate buffer pH9.6 with 1 mg/ml of MgCl_2_. The absorbance was recorded at 405 nm. Serum IgG subclasses were determined using anti–mouse IgG1, IgG2a or IgG2b alkaline-phosphatase conjugates (Sigma). To check that anti-IgG1, anti-IgG2a and anti-IgG2b were working properly, purified mouse IgG1 (clone CD28.2 [2 µg/mL]); mouse IgG2a (clone HIT3a [2 µg/mL]) and mouse IgG2b (clone G265-5 [2 µg/mL]), all from BD Pharmingen) were employed as positive controls. SDS-PAGE and Western blot of *L. intermedia* and *L. longipalpis* SGS was performed as described elsewhere [14]. Briefly, SDS-PAGE was performed by electrophoresis of SGS equivalent to 20 pairs of *L. intermedia* salivary glands (∼10 ug of protein) and SGS equivalent to 10 pairs of *L. longipalpis* salivary glands (∼10 ug of protein) in Bis-tris gels (4–12%, 1.0 mm) (Invitrogen). After transfer to nitrocellulose, the membrane was cut into strips, blocked overnight with blocking buffer (Tris HCl pH 8.0 NaCl 150 mM plus 5% non-fat milk), then incubated with mouse immune serum (1∶50 dilution) in blocking buffer, followed by the anti-mouse IgG alkaline phosphatase conjugate (1∶5000) (Promega). Alternatively, SDS-PAGE of *L. intermedia* SGS (100 pairs of *L. intermedia* salivary glands or ∼50 ug of protein) was performed on Bis-tris gels (4–12%, 1.0 mm×2D) (Invitrogen). After transfer to nitrocellulose, the membrane was blocked as above, incubated with human sera (1∶50 dilution), followed by the anti-human IgG alkaline phosphatase conjugate (1∶1000) (Sigma). Bands were visualized by adding alkaline phosphatase substrate (Promega).

### Analysis of the inflammatory immune response in the ear dermis

Following three intra-dermal inoculations with PBS or *L. intermedia* SGS in the right ear dermis, mice received a challenge injection with SGS (equivalent to 1 pair of salivary glands) in the left ear dermis. Twenty-four hours later, challenged ears were removed and fixed in 10% formaldehyde. Following fixation, tissues were processed, embedded in paraffin and 5 µm sections were stained with hematoxylin and eosin (H & E) and analyzed by light microscopy.

### Intracellular cytokine detection by flow cytometry

Reagents for staining cell surface markers and intracellular cytokines were purchased from BD Biosciences, San Diego, CA. Measurement of in vitro cytokine production was performed as described in de Moura et al. [20]. Briefly, cells were activated in the presence of anti-CD3 (10 µg/ml) and anti-CD28 (10 µg/ml) and were later incubated with Brefeldin A (Sigma) (10 µg/ml). Cells were blocked with anti-Fc receptor antibody (2.4G2) and were double stained, simultaneously, with anti-mouse surface CD4 (L3T4) and CD8 (53-6.7) conjugated to FITC and Cy-Chrome, respectively. For intracellular staining of cytokines, cells were permeabilized using Cytofix/Cytoperm (BD Biosciences) and were incubated with the anti-cytokine antibodies conjugated to PE: IFN-γ (XMG1.2), IL-4 (BVD4-1D11), and IL-10 (JES5-16E3). The isotype controls used were rat IgG2b (A95-1) and rat IgG2a (R35-95). Data were collected and analyzed using CELLQuest software and a FACSort flow cytometer (Becton Dickinson Immunocytometry System). The steady-state frequencies of cytokine positive cells were determined using lymph node cells from PBS-inoculated mice.

### Parasite culture, intradermal inoculation and lesion measurement

*L. braziliensis* promastigotes (strain MHOM/BR/01/BA788 [20]) were grown in Schneider medium (Sigma) supplemented with 100 U/ml of penicillin, 100 ug/ml of streptomycin, 10% heat-inactivated fetal calf serum (all from Invitrogen) and 2% sterile human urine. Two weeks following the last immunization with *L. intermedia* SGS, mice were challenged in the left ear dermis by inoculation of stationary-phase promastigotes (10^5^ parasites in 10 µl of saline)+SGS (equivalent to 1 pair of salivary glands). Lesion size was monitored weekly, for 16 weeks, using a digital calliper (Thomas Scientific).

### Parasite load estimate

Parasite load was determined using a quantitative limiting-dilution assay as described [22]. Briefly, infected ears and retromaxillar draining lymph nodes (LNs) were aseptically excised at two, four and 16 weeks post infection and homogenized in Schneider medium (Sigma). The homogenates were serially diluted in Schneider medium supplemented as before and seeded into 96-well plates containing biphasic blood agar (Novy-Nicolle-McNeal) medium. The number of viable parasites was determined from the highest dilution at which promastigotes could be grown out after up to two weeks of incubation at 25°C.

### Cytokine detection by ELISA

For measurement of in vitro cytokine production, single-cell suspensions of draining lymph nodes were prepared aseptically at two, four and 16 weeks post infection. The cells were diluted to 5×10^6^ cells/ml in RPMI 1640 supplemented with 2 mM L-glutamine, 100 U/ml of penicillin, 100 ug/ml of streptomycin, 10% fetal calf serum (all from Invitrogen) and 0.05 M 2-ME and dispensed into 96-well plates with *L. braziliensis* (ratio 1∶5, parasite:cells)+*L. intermedia* SGS (equivalent to 2.5 pair of salivary glands/ml) or *L. intermedia* SGS (equivalent to 2.5 pair of salivary glands/ml) alone. Cultures were incubated at 37°C in 5% CO_2_. Supernatants were harvested at 48 h and assayed for IL-4 or at 72 h and assayed for IFN-γ. Cytokine presence was determined by ELISA using commercial kits (BD Biosciences).

### Study population

Human sera used in the present study were obtained from an epidemiological survey conducted in a region endemic for American cutaneous leishmaniasis (Canoa, a rural village, located near Santo Amaro, Bahia, Brazil). Details of the area, patients and anti-*Leishmania* (Delayed Type Hypersensitivity) DTH skin test are described elsewhere [23]. Informed consent was obtained from patients or their guardians and all procedures were approved by the local Ethics Committee (CEP/MCO/UFA) and were conducted following recommendations outlined in the Helsinki Declaration. Briefly, after an outbreak of cutaneous leishmaniasis (CL) in Canoa, three groups of individuals were characterized: 1) Patients who developed CL; 2) Individuals who converted the DTH skin test to *Leishmania* antigen and who did not develop disease and 3) Individuals who had a negative anti-*Leishmania* DTH skin test. ELISA for detection of anti-saliva antibodies in these three groups was performed as described in [24]. Control sera were obtained from individuals residing in Salvador, BA, an area not endemic for leishmaniasis. These individuals showed negative responses to both anti-*Leishmania* sorology and anti-*Leishmania* DTH skin test. ELISA was performed as above. Briefly, microplates were coated with SGS diluted to 5 pairs of salivary glands/ml in coating buffer, wells were blocked, incubated overnight with human sera (1∶50 dilution), followed by alkaline phosphatase-conjugated anti-human IgG (1∶5000 dilution) .

### Statistical analysis

Data are presented as mean±standard error of the mean. The significance of the results was calculated by Mann Whitney or Kruskal-Wallis tests using Prism (Graph Pad Software) and P-values <0.05 were considered significant. To evaluate disease burden in mice, ear thickness of mice immunized with SGS and challenged with SGS+*L. braziliensis* was recorded weekly, for each individual mouse. The course of disease for experimental and control mice was plotted individually and the area under each resulting curve was calculated using Prism (Graph Pad Software). The significance of the results (area under curve obtained for each mouse immunized with SGS versus area under curve obtained for each mouse immunized with PBS) was calculated by Mann Whitney. In studies performed with human sera, significance of the results was calculated by Kruskal Wallis test followed by Bonferroni's Multiple Comparison Test.

## Results

### Immune response in *L. intermedia* saliva immunized BALB/c mice

**Figure 1:**
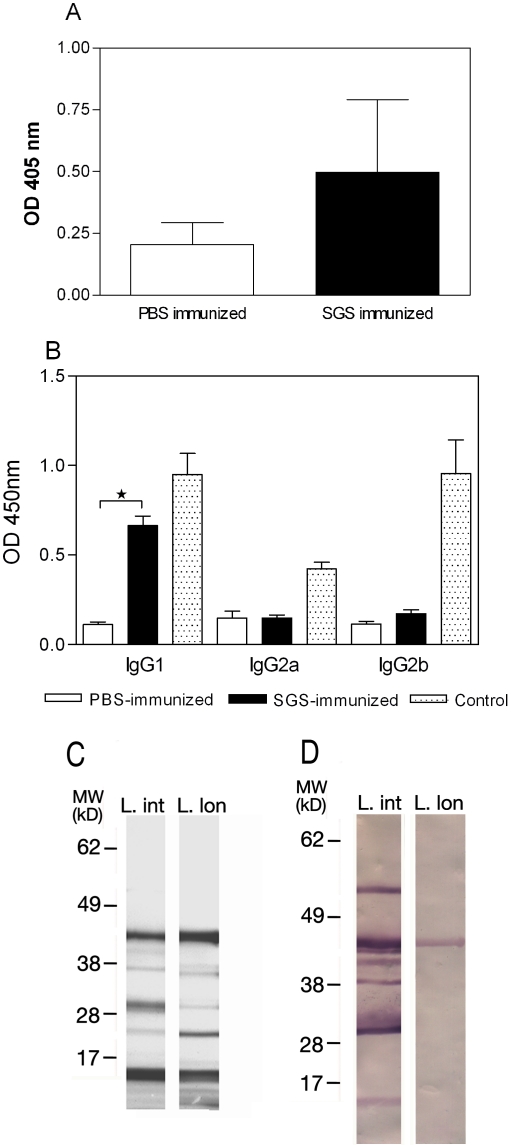
Anti-saliva antibody response following immunization of BALB/c mice with *Lutzomyia intermedia* SGS. BALB/c mice (3–5 per group) received 3 inoculations of PBS (open bars) or SGS (closed bars). (A) ELISA was performed with sera from mice inoculated with PBS (open bars) or SGS (closed bars). (B) IgG subclasses present in immune sera were determined by ELISA using IgG1, IgG2a and IgG2b conjugates. Purified IgG1, IgG2a and IgG2b (dotted bars) were employed as control IgG subclass (positive controls). Bars represent the means and standard errors of the means from three independent experiments (*p<0.05). (C) Commassie blue-stained SDS-PAGE gel after electrophoresis of *L. intermedia* (L.int) and *L. longipalpis* (L.lon) SGS. The numbers represent the position of the mol wt markers. (D) Western blots of the gels showed in C, probed with sera from *L. intermedia* SGS immunized mice.

Sera from immunized mice obtained two weeks after the last immunization were able to recognize *L. intermedia* SGS by ELISA (Fig. 1A) and, importantly, the presence of IgG1 was significantly higher in mice *L. intermedia* SGS-immunized when compared to PBS-immunized mice. Similar differences were not observed when we compared IgG2a and IgG2b levels in SGS and PBS-immunized mice (Fig. 1B). Western blot confirmed that immune sera specifically recognized the majority of bands seen in *L. intermedia* SDS-PAGE profiles (Fig. 1D and 1C, respectively). When *L. intermedia* SGS immune sera were probed against *L. longipalpis* SGS, the only protein recognized was a protein of approximately 45 kDa, a protein with similar mobility to the yellow related protein from *L. longipalpis* (Fig. 1D).We can speculate that, although the salivary gland protein profiles of both sand fly species appear similar (Fig. 1C), their antigenic properties are different (Fig. 1D).

**Figure 2:**
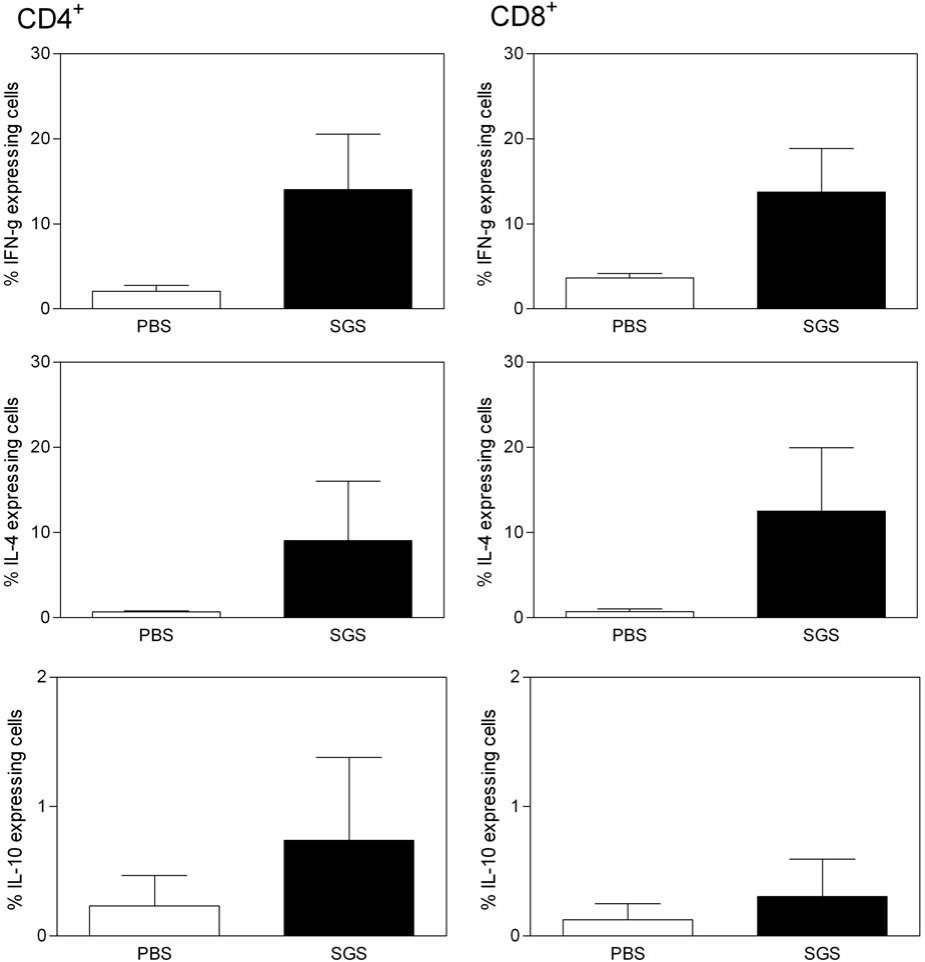
Intracellular cytokine production by CD4 and CD8 T cells following immunization of BALB/c mice with Lutzomyia intermedia SGS. BALB/c mice (3–5 per group) received 3 inoculations of PBS (open bars) or SGS (closed bars). Fifteen days after the last inoculation, draining lymph nodes were pooled and cells were preincubated with Brefeldin A for four hours before being stained. Data represent the percentages of cells with signals for the particular cytokine that were greater than the background signals established using isotype controls. Bars represent the means and standard errors of the means of one experiment, representative of three independent experiments.

A mixed cytokine response, with the presence of both IFN-γ and IL-4 was observed in immunized animals (Fig. 2A). These cytokines were detected within CD4+ (left panel) and CD8+ T cells (right panel), at comparable levels. IL-10 expression was also detected in these two cell populations, albeit in a lower frequency when compared to IFN-γ and IL-4.

**Figure 3:**
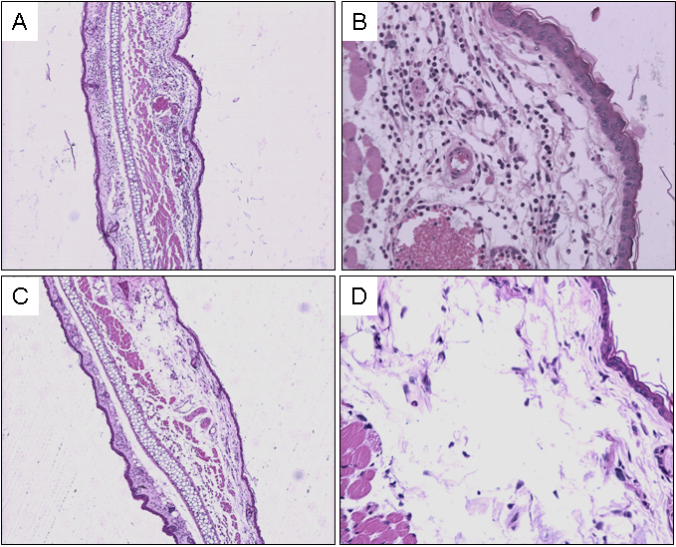
Histological aspects in animals immunized with *L. intermedia* SGS. Mice received three inoculations with SGS (A and B) or PBS (C and D). Two weeks after the last inoculation, mice received a challenge with SGS in the contra-lateral ear. Tissue sections were obtained 24 h after challenge and stained with hematoxylin and eosin and analyzed under a light microscope. Magnifications are ×40 (A and C) and ×200 (B and D).

*L. intermedia* SGS also induced an important inflammatory response in immunized mice. Injection of SGS following immunization with SGS elicited, at 24 h post SGS injection, an inflammatory infiltrate comprised of numerous polymorphonuclears (PMNs) and few mononuclear cells (Fig. 3A and B). Edema and vascular congestion were also noted. At 48 h post challenge injection with SGS, the infiltrate was more pronounced, with higher numbers of PMNs and mononuclear cells and accentuated myositis (not shown). In mice which received PBS and were later challenged with SGS, there was edema formation with rare inflammatory cells at 24 h (Fig. 3C and D). This effect was decreased at 48 h (data not shown).

### *L. braziliensis* infection in *L. intermedia*-saliva immunized mice

**Figure 4:**
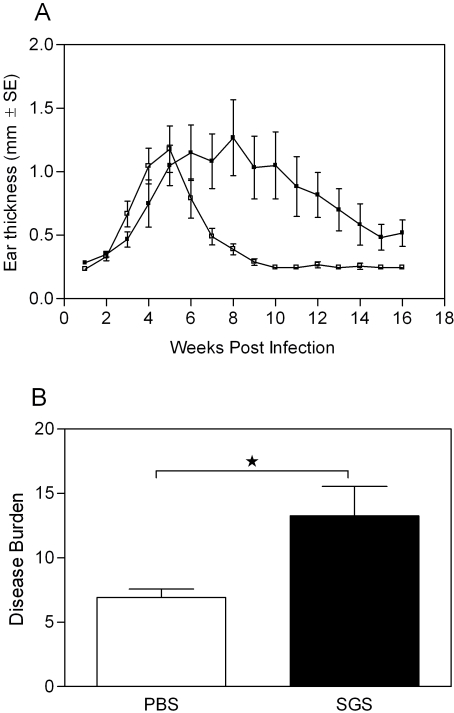
Lesion development in *L. intermedia* SGS immunized BALB/c mice following infection with *L. braziliensis*+SGS. BALB/c mice (3–5 per group) were inoculated three times in the right ear with *L. intermedia* SGS (closed bars) or with PBS (open bars) and were challenged in the left ear with *L. braziliensis* plus *L. intermedia* SGS (equivalent to 1 pair of salivary gland). (A) The course of lesion development was monitored weekly and bars represent the means and standard errors of the means from three independent experiments. (B) Disease burden in mice inoculated with PBS or SGS and challenged with SGS+*L. braziliensis*. The areas contained underneath the curves obtained in (A) for each experimental and control mouse were compared. (*p<0.05).

We then examined whether immunization with *L. intermedia* saliva altered the course of *L. braziliensis* infection in BALB/c mice, using an intradermal experimental model of infection [20]. Surprisingly, pre-immunization with *L. intermedia* SGS did not protect against lesion development. As shown in Fig. 4A, the onset of lesion development in PBS-inoculated mice was at three weeks post infection, peaking at five weeks and resolving, spontaneously, by returning to normal ear thickness after ten weeks. In *L. intermedia* SGS immunized mice, however, dermal lesions appeared later, at five weeks post infection and peaked at eight weeks post infection (Fig. 4A). From then on, lesions slowly declined and resolved at 16 weeks post infection, although ear thickness never returned to normal levels (0.2–0.3 mm). No significant differences in ear thickness, between both groups, were observed throughout infection. However, two weeks following infection, SGS–immunized mice showed a slight inflammatory infiltrate with a discrete increase in PMNs (data not shown). Importantly, a significant difference (p<0.05) was observed when we evaluated disease burden in SGS and PBS-immunized mice (Fig. 4B). Disease burden was calculated by weekly measure of ear thickness and by comparison of the area under the resulting curves, as explained under statistical analysis. As shown in Fig. 4B, dermal lesions persisted for a longer period of time in SGS-immunized mice indicating that disease burden is more pronounced in these mice when compared to PBS-immunized mice.

### Parasite load in the ear dermis and draining lymph nodes of *L. intermedia* saliva immunized mice challenged with *L. braziliensis*

**Figure 5:**
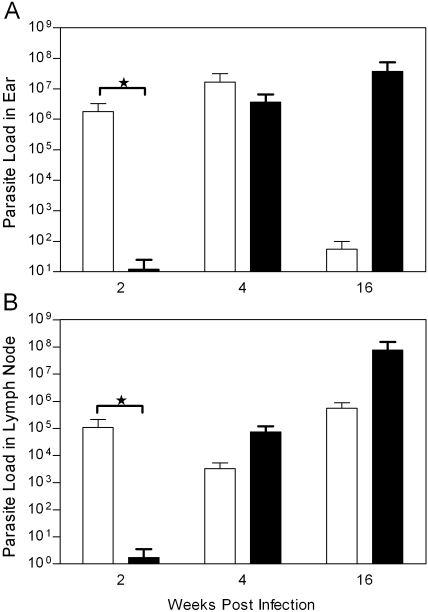
Parasite load estimate in BALB/c mice immunized with *L. intermedia* SGS or PBS and challenged with *L. braziliensis* in the presence of *L. intermedia* SGS. BALB/c mice (3–5 per group) were inoculated three times with PBS (open bars) or SGS (closed bars). Two weeks after the last inoculation, mice were challenged with 10^5^ *L. braziliensis*+SGS (equivalent to 1 pair of salivary glands/animal). Ear (A) and draining lymph node (B) parasite loads were determined at two, four and 16 weeks post infection via a limiting dilution assay. Bars represent the means and standard errors of the means from three independent experiments. (*p<0.05)

We then examined whether there was a correlation between lesion development and parasite replication in *L. intermedia* saliva immunized mice. As shown in Fig. 5, two weeks after challenge, parasite load in the ear dermis and draining lymph nodes was significantly lower in saliva immunized mice (Fig. 5A and B). Four weeks after challenge, parasite load was similar in both groups of animals, in the two compartments analyzed, which correlated with the similar lesion sizes (Fig.4A). Later, 16 weeks following challenge, mice immunized with *L. intermedia* SGS displayed a significantly higher parasite load in the ear (Fig. 5A) whereas no significant differences were observed in the draining lymph nodes of both groups (Fig. 5B). At this same time point, however, there was no significant difference in ear thickness in both groups of mice.

### Immune response in *L. intermedia* saliva immunized mice challenged with *L. braziliensis*

**Figure 6:**
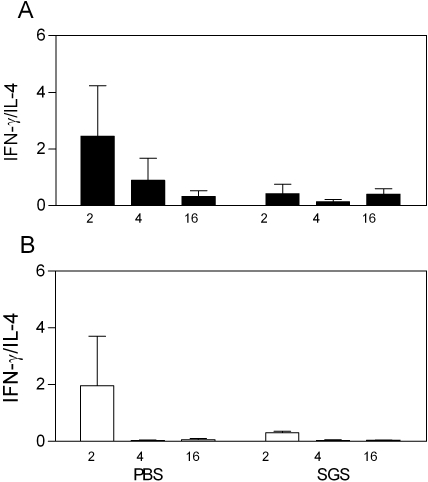
IFN-γ to IL-4 ratio in BALB/c mice inoculated with *L. intermedia* SGS or PBS and challenged with *L. braziliensis* in the presence of *L. intermedia* SGS. Mice were inoculated three times with PBS or SGS. Two weeks after the last inoculation, mice were challenged with 10^5^ *L. braziliensis*+SGS (equivalent to 1 pair of salivary glands/animal). Two, four and 16 weeks post challenge, draining lymph node cells were collected and stimulated with *L. braziliensis*+SGS (A) or with SGS alone (B). Culture supernatants were collected and cytokine presence was determined by ELISA. The data represent the means and standard errors of the means from three independent experiments, each performed with five mice per group.

Next, we examined the immune response in mice immunized with *L. intermedia* saliva and challenged with *L. braziliensis* plus SGS. Two weeks following infection, the IFN-γ to IL-4 ratio is two-fold higher in animals inoculated with PBS when compared to mice immunized with SGS (Fig. 6). This higher IFN-γ production in relation to IL-4 was observed following in vitro stimulation with either *L. braziliensis*+SGS (Fig. 6A) or SGS alone (Fig. 6B). Later on, the IFN-γ to IL-4 ratio in PBS inoculated mice, decreased steadily, correlating with the decrease in ear thickness (Fig. 4A) and in parasitemia at the inoculation site (Fig. 5A). Overall, SGS immunized mice produced much less IFN-γ in relation to IL-4 after stimulation with either *Leishmania* plus SGS or after stimulation with SGS alone, which can be correlated with the higher disease burden (Fig. 4B) and the constant increase in parasite load (Fig. 5A).

### Humoral immune response against *L. intermedia* saliva in an endemic area for Cutaneous Leishmaniasis

**Figure 7:**
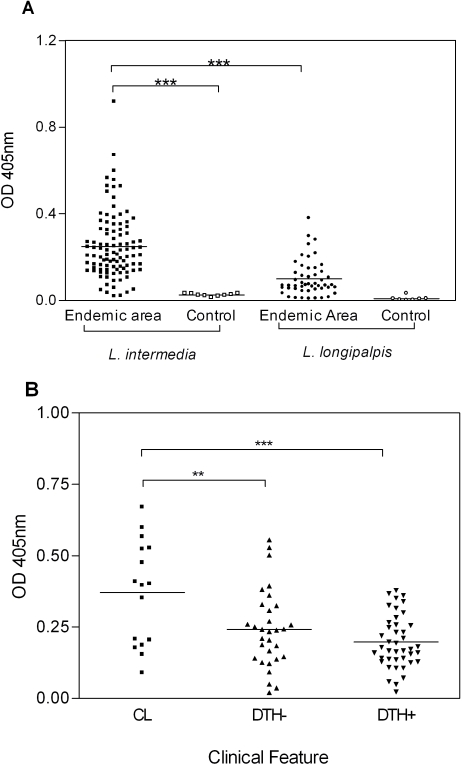
Human serum IgG response against SGS. (A) ELISA was performed against *L. intermedia* and *L. longipalpis* SGS using human sera were from individuals from a CL endemic area (n = 100) or from control individuals (n = 10). (B) ELISA was performed against *L. intermedia* SGS with human sera from CL individuals (n = 16) or from healthy individuals with either a negative (n = 42) or a positive (n = 32) anti-*Leishmania* DTH skin test. The ordinate represents the absorbance of the ELISA reaction of these sera against SGS. The symbols indicate results obtained with each serum tested and lines represent their median values (***p<0.001).

**Figure 8:**
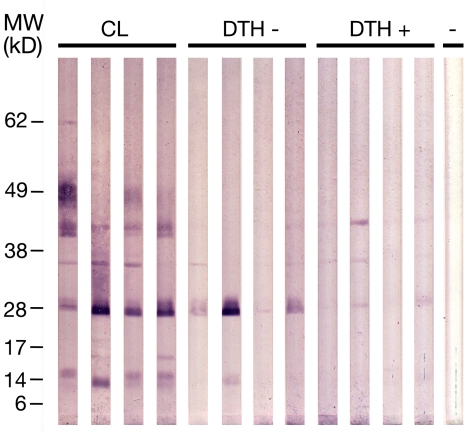
Western blot of *Lutzomyia intermedia* salivary proteins recognized by sera from exposed individuals. Western blot was performed with human sera from CL individuals (n = 4) or from healthy individuals with either a negative (n = 4) or a positive (n = 4) anti-*Leishmania* DTH skin test. The numbers represent the position of the mol wt markers. (-) Serum from control individual.

Data obtained in mice immunized with *L. intermedia* saliva and later challenged with *L. braziliensis* showed that, in this case, saliva immunization failed to protect against a challenge infection with *L. braziliensis*. Although immunization with *L. intermedia* saliva delayed lesion appearance, lesions persisted for longer periods when compared to PBS inoculated mice. Upon this finding, we then asked whether human antibody response to *L. intermedia* saliva could be used to monitor exposure to sand fly and, possibly, be used as a marker of disease. In order to do so, we first investigated the anti-*L. intermedia* humoral immune response in individuals living in an endemic area for cutaneous leishmaniasis (CL). As shown in Fig. 7A, CL endemic area individuals possessed significantly higher anti-*L. intermedia* SGS IgG levels when compared to control sera (sera obtained from individuals residing in a non-endemic area). When sera from a CL endemic area were probed against *L. longipalpis* SGS, IgG levels were significantly lower when compared to IgG levels against *L. intermedia* SGS, illustrating the specificity of the anti-saliva immune response. We then selected, among individuals from the endemic area with positive anti-*L. intermedia* SGS IgG response (Fig. 7A), a subgroup of patients with active lesion at the time of serum collection (CL), a subgroup of individuals with positive anti-*Leishmania* DTH skin test and a subgroup with negative DTH skin test. Sera from these three subgroups were again probed against *L. intermedia* SGS. As shown in Fig. 7B, CL patients displayed a significantly higher IgG immune response against saliva when compared to individuals with positive DTH. This was also observed when comparing CL patients and DTH negative individuals. These results show that humoral immune response to *L. intermedia* SGS is a marker of disease in CL. Western blot analysis (Fig. 8) showed that sera from CL individuals displayed the strongest response against *L. intermedia* SGS, when compared to DTH− and DTH+ individuals. This strong response was observed particularly for the 62 kD, 49 kD, 45 kD, 36 Kd, 28 kD and 14 kD bands. Sera from DTH− individuals, on the other hand, recognized, preferentially, the 28 kD and, in some cases, the 14 kD and 36 kD bands. Last, sera from DTH+ individuals showed the weakest response, when compared to CL and DTH− individuals. Nonetheless, these sera were able to detect, for some individuals, the 45 kD, 36 kD and 28 kD bands. Sera from controls individuals (-) did not recognize any of the salivary proteins.

## Discussion

The results presented herein show that *L. intermedia* SGS shifted the immune responses to *L. braziliensis* to type 2 and immunization of BALB/c mice with SGS led to enhanced *L. braziliensis* infection. Additionally, induction of this distinctive role played by *L. intermedia* saliva comes from data obtained with patients with active CL who presented higher anti-*L. intermedia* SGS antibody titers when compared to exposed individuals with positive anti-*Leishmania* DTH.

Initially, we investigated the immunogenic properties of *L. intermedia*, the main vector of *L. braziliensis* in Brazil. We observed that mice immunized with *L. intermedia* SGS developed a specific humoral immune response as shown by ELISA and Western blot. The main subclass present in immune sera was IgG1. In the absence of IgG2a, this is indicative of a preferential type 2 immune response, knowingly associated with susceptibility to leishmaniasis (revised in [25]. The presence of IgG1 was also observed upon natural exposure of mice to *L. longipalpis* bites [5] and upon immunization with *P. ariasi* SGS [26]. In an area endemic for visceral leishmaniasis, exposed individuals showed a mixed composition of IgG1 and IgE antibodies to *L. longipalpis* saliva, suggesting a mixed type1/type 2 response [27]. This type of response has also been detected herein as shown by the presence of both IFN-γ and IL-4 in cell culture supernatants from *L. intermedia* SGS immunized mice.

Specificity of the humoral immune response was tested by probing immune sera with *L. longipalpis* SGS, this species being the vector of *L. chagasi*, the causative agent of American visceral leishmaniasis. Interestingly, SDS-PAGE profiles of salivary proteins from both sand flies showed bands migrating at similar molecular weights. However, *L. intermedia* immune sera did not recognize any proteins present in *L. longipalpis* SGS with the exception of the ∼45 kDa protein. The species-specificity of sand fly salivary gland components has been examined elsewhere [28],[29]. Thiakaki et al. [30] showed that mice exposed to bites of three sand fly species developed antibodies specific to the different saliva antigens, indicating the specificity of anti-saliva immune responses. Indeed, results from our laboratory have shown that salivary content similarity between *L. intermedia* and *L. longipalpis* is low (de Oliveira et al., manuscript in preparation) corroborating the lack of antibody of cross-reactivity and emphasizing the uniqueness of the sand fly saliva antigens.

Surprisingly, immunization with *L. intermedia* SGS did not confer any protection against *L. braziliensis* development: SGS immunized mice developed larger lesions which were maintained for a longer period when compared to PBS inoculated mice. As observed before in *L. braziliensis* infected mice [20], parasites were able to persist in draining lymph nodes, regardless of lesion healing. The lack of protection against a challenge infection in SGS immunized mice can therefore be correlated with the low IFN-γ to IL-4 ratio observed throughout the infection period, an effect not observed in PBS inoculated mice. In the latter, disease burden was significantly lower, indicating their ability to mount and sustain a balanced immune response. Initially, SGS immunized mice showed a significantly lower parasite burden after challenge with parasites plus saliva. It is possible that this early control in parasitemia may be exerted by inflammatory cells (mono and polymorphonuclear cells) that are recruited following stimulation with saliva. This early control, however is not maintained since parasite multiplication was clearly observed, probably as a result of the pathogen-favorable adaptative immune response that is developed in SGS immunized mice. It has been shown that macrophages phagocytose apoptotic neutrophils infected with *L. major* [31] and also that coinjection of dead neutrophils amplified *L. major* replication in vivo in BALB [32]. It is tempting to speculate that the rise in parasite burden observed later on is related to similar effects exerted by the PMNs recruited following immunization with *L. intermedia* saliva and this hypothesis is under current investigation.

In the *L. major* model, where immunization with *P. papatasi* SGS leads to protection against challenge, it is postulated that SGS immunization precludes the production of type 2 cytokines [8],[12]. It was also shown that immunization with SP15, antigen present in *P. papatasi* saliva, leads to the development of an anti-saliva DTH response [14], which was also associated with protection. Herein, immunization with *L. intermedia* SGS did not inhibit the production of type 2 cytokines or promoted the development of a classical DTH reaction, with the characteristic presence of macrophages and lymphocytes. Accordingly, immunization with *L. intermedia* SGS is favoring *L. braziliensis* establishment and persistence in BALB/c mice.

In human visceral leishmaniasis, individuals with positive anti-*Leishmania* cellular immune response (a putative marker of protection against *L. chagasi*) have increased anti *L. longipalpis* saliva IgG levels when compared to individuals with positive anti-*Leishmania* humoral immune response (a marker of *L. chagasi* infection) [24],[27]. In the present study, however, we found an opposite correlation: a higher anti-*L. intermedia* saliva immune response was observed in CL patients whereas individuals from the endemic area with a positive DTH skin test against *Leishmania* showed a lower IgG response anti saliva. It is tempting to speculate that the detrimental effects of sensitization with *L. intermedia* saliva observed in mice have a parallel in individuals exposed in the endemic area. Rohousosva et al., [29] observed that *L. tropica* patients possessed significantly higher anti-*P. sergenti* saliva IgG levels when compared to healthy individuals from the same place. In this work, authors suggested that higher anti-saliva IgG levels may reflect a more frequent exposure to vector bites and, therefore, higher probability of *L. tropica* transmission. In our study, we also observed a higher anti-*L. intermedia* SGS humoral response in individuals from a CL endemic area when compared to control individuals which may also reflect a more frequent exposure to vector bites. More importantly, however, we found that, among individuals from the endemic area, anti-saliva IgG responses were significantly lower in both DTH+ (individuals who developed an anti-*Leishmania* cellular immune response when exposed to infected sand flies) and in DTH− (individuals who did not develop an anti-*Leishmania* cellular immune response), when compared to the anti-saliva response in CL patients. It would be most interesting to determine whether DTH− individuals either convert to DTH+ or develop CL and whether the anti-*L. intermedia* saliva humoral response, in these cases, increases or decreases, respectively. Nonetheless, the current results enable us to hypothesize that, for CL caused by *L. braziliensis*, a higher anti-saliva humoral immune response could be used as a marker of risk for *Leishmania* transmission, a finding reported for the first time in New World cutaneous leishmaniasis.

Characterization of the immune response to sand fly saliva has proven useful in understanding how the molecules present therein modulate host's immune response. In the case of *P. papatasi* and *L. major*, a vaccine candidate has been identified and this type of study now constitutes a major area of research in the development of control measures against leishmaniasis [26]. However, we have shown that this is not necessarily applicable to other vector/parasite systems such as *L. braziliensis/L. intermedia*. Our study was conducted with inoculation of SGS and we cannot, presently, exclude that the effects observed in the mouse model are due to an immune response against saliva alone and not against a mixture of saliva and structural components of the salivary gland or even LPS. Regarding the latter, *L. intermedia* SGS preparations were unable to induce TNF-α production following stimulation of human monocytes (data not shown) and such effect is probably due to the very low concentrations of LPS (<0.1 ng/ml, below the detection limit of the *Limulus* amebocyte assay). Indeed, it would be very interesting to determine the outcome of *L. braziliensis* infection in mice sensitized to *L. intermedia* sand fly bites. However, rearing of *L. intermedia* sand flies under laboratory conditions has proven a challenging process. To date, there has been very little work on transmission with *L. braziliensis* parasites, especially using the sand fly vectors, and experimental transmission of *Viannia* parasites by infected sand fly bite has not been achieved [33]. However, it has been shown that, regarding the use of SGS, mice develop a strong DTH response after a double exposure to the equivalent of 0.2 pairs of salivary glands, inoculated intradermally in the ear, and that a similar cellular infiltrate is mobilized to the skin following sand fly bites [34]. Moreover, it was also shown that there is little difference in the composition of sand fly saliva and SGS since major protein components of SGS are present in the salivary contents [35], confirming that SGS is an appropriate source of antigen to mimic natural exposure to sand fly saliva. Nonetheless, since vector based vaccines are an important alternative envisaging disease control, we are currently investigating whether immunization with individual *L. intermedia* salivary components is capable of inducing protection.
